# Isolation and characterization of extracellular vesicles for clinical applications in cancer – time for standardization?

**DOI:** 10.1039/d0na00676a

**Published:** 2021-02-16

**Authors:** Nikki Salmond, Karla C. Williams

**Affiliations:** University of British Columbia, Faculty of Pharmaceutical Sciences Vancouver V6T 1Z3 Canada karla.williams@ubc.ca

## Abstract

Extracellular vesicles (EVs) are nanometer sized lipid enclosed particles released by all cell types into the extracellular space and biological fluids *in vivo*, and into cell culture media *in vitro*. An important physiological role of EVs is cell–cell communication. EVs interact with, and deliver, their contents to recipient cells in a functional capacity; this makes EVs desirable vehicles for the delivery of therapeutic cargoes. In addition, as EVs contain proteins, lipids, glycans, and nucleic acids that reflect their cell of origin, their potential utility in disease diagnosis and prognostication is of great interest. The number of published studies analyzing EVs and their contents in the pre-clinical and clinical setting is rapidly expanding. However, there is little standardization as to what techniques should be used to isolate, purify and characterize EVs. Here we provide a comprehensive literature review encompassing the use of EVs as diagnostic and prognostic biomarkers in cancer. We also detail their use as therapeutic delivery vehicles to treat cancer in pre-clinical and clinical settings and assess the EV isolation and characterization strategies currently being employed. Our report details diverse isolation strategies which are often dependent upon multiple factors such as biofluid type, sample volume, and desired purity of EVs. As isolation strategies vary greatly between studies, thorough EV characterization would be of great importance. However, to date, EV characterization in pre-clinical and clinical studies is not consistently or routinely adhered to. Standardization of EV characterization so that all studies image EVs, quantitate protein concentration, identify the presence of EV protein markers and contaminants, and measure EV particle size and concentration is suggested. Additionally, the use of RNase, DNase and protease EV membrane protection control experiments is recommended to ensure that the cargo being investigated is truly EV associated. Overall, diverse methodology for EV isolation is advantageous as it can support different sample types and volumes. Nevertheless, EV characterization is crucial and should be performed in a rigorous manor.

## Abstract 2

Clinical applications for extracellular vesicles (EVs): tumor derived EVs represent a non-invasive testing platform for cancer detection and engineered EVs represent a therapeutic strategy for cancer treatment.

## Graphical abstract

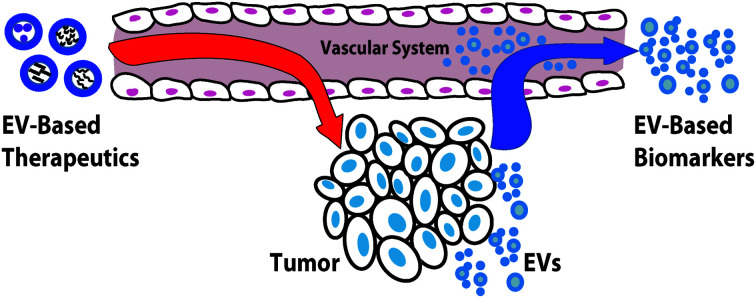

## Introduction to extracellular vesicles

### EV biogenesis

**Fig. 1:**
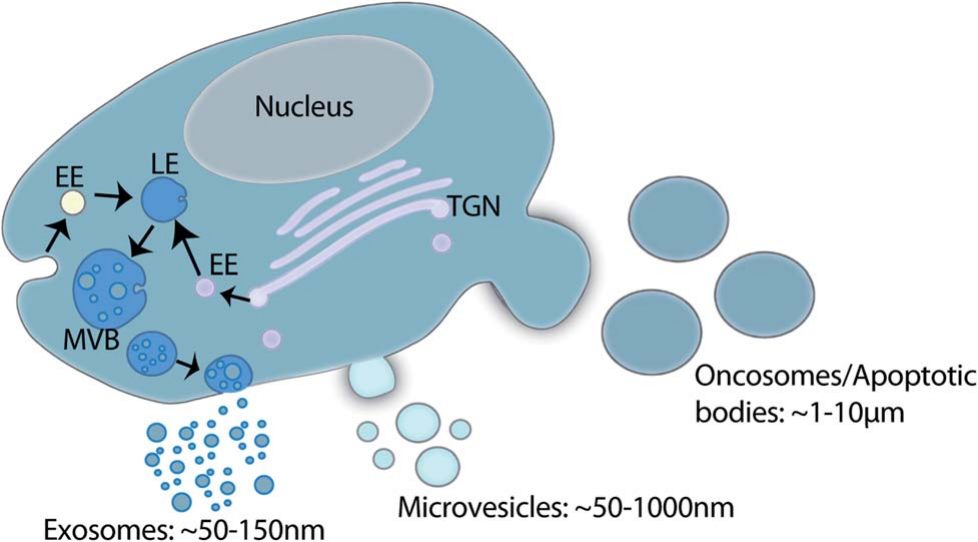
Schematic of EV family and biogenesis. Exosome biogenesis takes place within the endosomal system whereby the inward budding of the endosomal membrane forms intraluminal vesicles within the late endosomes (LE). The LE contains cargo from multiple vesicular trafficking routes (EE: early endosome; TGN: *trans*-Golgi network). The resultant multivesicular body (MVB) fuses with the plasma membrane to release intraluminal vesicles into the extracellular environment as exosomes. Microvesicles, oncosomes and apoptotic bodies all shed directly from the plasma membrane. Collectively this family of vesicles is termed extracellular vesicles.

Extracellular vesicles (EVs) are lipid membrane enclosed nano-sized particles released into the extracellular environment and biological fluids by virtually every cell line and cell type. The term EV is used to encompass a large family of vesicles including, exosomes, microvesicles, oncosomes and apoptotic bodies.^1^ Exosome (∼30–150 nm) biogenesis occurs within the late endosome through inward membrane budding and fission which generates intraluminal vesicles and creates a structure classically described as the multivesicular body (MVB). Fusion of the MVB at the plasma membrane releases intraluminal vesicles into the extracellular environment as exosomes.^1–3^ Microvesicle (∼50–1000 nm), oncosome (∼1–10 μm), and apoptotic body (∼1–5 μm) biogenesis occurs *via* direct membrane budding and fission at the plasma membrane.^1,4^ Oncosome biogenesis has been associated with cancer cells,^5–7^ and apoptotic bodies are released by cells undergoing apoptosis (Fig. 1).^8,9^

### EV content and function

EVs contain ribonucleic acid (RNA),^10–13^ deoxyribonucleic acid (DNA),^14,15^ proteins,^6,16^ lipids,^17–20^ metabolites^21,22^ and glycans^23–25^ from the cell of origin. Although originally thought to be trash containers for cellular waste removal,^26^ work over the last decade has highlighted the diverse and important functions of EVs in cell–cell communication, maintaining homeostasis, and in multiple pathological conditions including cancer.^27,28^ After release into the extracellular space, or into biological fluids (*i.e.* blood, urine, saliva, breast milk),^29–31^ EVs are taken up by recipient cells and deliver their functional protein and nucleic acid contents to alter the recipient cell phenotype.^27^ This exchange of information between cells not only occurs locally between neighboring cells, but also with cells at distant sites of the body after transport in biological fluids such as blood.^32–36^ EVs have a well-established role in physiological processes such as coordinating the immune response,^37^ coagulation^38^ and angiogenesis.^39^ Just as EVs are effective in delivering cargo to recipient cells to support healthy homeostasis, EVs from diseased cells also deliver their contents to recipient cells promoting multifarious phenotypic changes locally and regionally at distant organs. EVs have been found to have important roles in neurodegenerative,^40^ cardiovascular,^41^ and inflammatory^42^ disease states amongst many.

### EV function in cancer

A rapidly expanding field of EV research is that of EVs in cancer biology. EVs released by cancer cells have demonstrated roles in all stages of cancer progression and metastasis. However, as the focus of this review is on EV developments in the clinical setting we will only briefly describe some highlights on EV functions in cancer and direct readers to comprehensive reviews by others detailing EV-based cell-to-cell communication,^28,43,44^ immune modulation,^45–48^ drug resistance,^49^ and the pre-metastatic niche in cancer.^33^

The first report suggesting a role for EVs in neoplastic cell function was published in 1981.^50^ Following this, some of the first discoveries on EV function in cancer identified immunomodulatory roles for EVs. In 1998, a pivotal study by Zitvogel, L. *et al.* isolated tumor antigen-presenting exosomes from dendritic cells pulsed with tumor antigens and demonstrated their immunostimulatory effect which decreased tumor growth, and in some instances even resulted in tumor eradication.^51^ Furthermore, it was shown that tumor-derived EVs transfer tumor antigens to dendritic cells and stimulate antitumor effects.^52^ These early studies introduced the field to the possibility that EVs could be used in a therapeutic capacity for cancer treatment. Whilst the immunostimulatory properties of EVs can increase tumor recognition, studies focused on cancer cell derived EVs have also detailed potent immunosuppression of T-cells,^53,54^ Natural Killer cells,^55^ promotion of myeloid suppressor cell differentiation^56^ and pro-tumorigenic differentiation of macrophages.^57^ Taken together, all these studies highlight the complex and diverse actions of EVs.

Cancer cell derived EVs not only modulate the immune system but also support the transfer of EV nucleic acid and protein contents between neoplastic cells to promote tumorigenic phenotypes. For example, it has been observed that mutant Epidermal Growth Factor Receptor (EGFR) protein can be transferred to neighboring cells *via* cancer cell derived EVs and promote a tumorigenic phenotype in recipient cells.^6^ Additionally, EVs released by glioblastoma cells deliver protein and RNA cargoes to recipient cells to promote angiogenesis and tumorigenesis.^12^ Cancer cell EVs support not only local cell-to-cell communication, but also act on cells at distant sites. This was elegantly shown by Zomer, *et al.* (2015) using intravital *in vivo* imaging to show the transfer of cre recombinase containing EVs from tumor cells to less malignant recipient cre reporter cells both locally and systemically. Not only was cre transferred in a functional capacity, but less malignant recipient cells started to display increased migratory and metastatic phenotypes demonstrating EV mediated transfer of tumorigenic phenotypes between different cell populations.^32^ Additionally, melanoma derived EVs educate bone marrow progenitor cells towards a pro-metastatic phenotype, and promote vascular leakiness at pre-metastatic sites, thus, playing a role in pre-metastatic niche formation.^36^ Pancreatic cancer cell derived EVs have been found to promote fibrotic pre-metastatic environment formation in the liver through education of kupffer cells and recruitment of macrophages.^35^ Interestingly, EV priming of the metastatic niche is thought to depend upon specific integrin expression on cancer cell derived EVs; integrins appear to impart a tropism of EVs to specific extracellular matrices within organs and can be predictive of metastatic site.^35^ These studies are a few of many which highlight the functional role of cancer derived EVs and support the notion of a unique molecular profile which could potentially be used for disease detection, monitoring and prognosis.

### EVs as non-invasive biomarkers in cancer

**Fig. 2:**
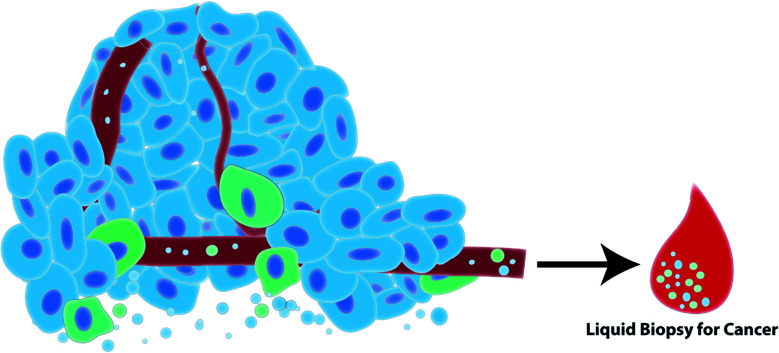
Tumor cells (blue) and immune cells (green) in the tumor microenvironment release EVs into the extracellular space and into biological fluids such as blood. Such biological fluids can be harvested in a minimally-invasive liquid biopsy to diagnose and prognosticate cancer.

Growing interest in the use of EVs for biomedical research has led to an explosion of EV-based publications aiming to develop liquid biopsies for cancer. EVs act as windows of information about the cell from which they derived in their nucleic acid, protein and lipid signatures. In cancer, cells often exhibit unique nucleic acid/protein/lipid profiles that should be reflected in the EVs that those cells release. For example, proteomic analysis identified epithelial mesenchymal transition (EMT) associated proteins in EVs released by metastatic bladder cancer cells but not non-metastatic bladder cancer cells.^58^ Additionally, alterations and mutations in cancer cell DNA can be detected in the EVs released by cancer cells.^14,15,59^ This suggests that interrogation of EV content can be exploited for cancer diagnosis and prognostication. EVs can be readily detected in biological fluids such as blood, urine and saliva which further supports the use of EVs as ideal biomarker candidates in the development of minimally invasive testing platforms for cancer (Fig. 2).^29–31,60^ Cargo stability is another advantage of EVs. EVs are enclosed by a lipid bilayer which protects its nucleic acid and protein contents from degradation in the circulation and during isolation and storage.^61,62^ Importantly, these attributes support the retrospective isolation and analysis of EVs from biobanked biological samples in pre-clinical biomarker discovery phases of research.

### EVs as therapeutics in cancer

Autologous and HEK293 cell line derived EVs are seemingly immunologically and toxicologically inert.^63,64^ These properties alongside the ability of EVs to be taken up by and deliver functional cargoes to recipient cells locally and at distant sites in the body, make EVs attractive candidates to use as therapeutic delivery vehicles. This field of research explores EV loading with therapeutic cargoes by engineering cell lines or through EV manipulation post-isolation. Whilst most clinical studies use autologous cell derived EVs, pre-clinical studies use cell line derived EVs for proof of principle and concept development. In order to use EVs for clinical applications the isolation and characterization of EVs needs to be carefully planned and controlled.

In this review, we assess the different EV isolation and characterization techniques employed in biomarker and therapeutic development studies and discuss their utility in pre-clinical and clinical studies.

## Isolation and characterization of extracellular vesicles

### EV isolation

For EV use in clinical applications, the isolation strategy needs careful consideration since it directly effects the EV population isolated and therefore the study outcome. The isolation and purification of EVs from biological fluids and cell culture supernatants can be achieved using several different techniques. Important considerations in method selection include the fluid from which EVs are to be isolated (cell culture supernatant, blood, urine *etc.*), the volume of fluid from which EVs will be isolated, and the desired EV purity. EV purity is of critical importance for use as a clinical therapeutic to ensure engineered EVs are pure and free of contaminating proteins and nucleic acids that could have a negative impact upon clinical administration. Additionally, separation of EVs away from other proteins and nucleic acids ensures that biological effects of therapeutic vesicles are attributed to EV enclosed/associated cargoes and not co-purified contaminants. However, for clinical biomarker studies, depending on what is being studied EV purity may be less of a concern. Analysis of select biomarkers *via* sequencing, Enzyme Linked Immunosorbent Assay (ELISA), or nanoscale flow cytometry would be less dependent on purity and more concerned with quantity. On the other hand biomarker discovery studies would require a high level of purity and thorough characterization of EVs before proceeding to validation studies and clinical applications.

A classical and commonly used EV isolation technique is differential ultracentrifugation. Often considered the “gold standard” for EV isolation, this technique uses increasing centrifugation force to remove cells and debris from cell culture supernatant or biological fluid (300*g* & 2500*g*), pellet large EVs (10 000*g*), and finally small EVs (100 000*g*/200 000*g*).^65^ Results from the literature, however, bring in to question the reproducibility of studies using this isolation method. Potential causes for this include rotor use (swing bucket *versus* fixed angle),^66^ sample viscosity,^67^ and tube *k*-factor.^66^ EV preparations using high centrifugal forces can contain contaminating particles from protein aggregation and other contaminants. Ultracentrifugation alone cannot remove contaminating lipoproteins from biological samples, such as blood, unless used in conjunction with a gradient and/or other chromatography techniques.^68–70^ Sucrose or iodixonal density gradients can be used to separate EVs from contaminating proteins, whereby solutions of increasing concentrations of sucrose or iodixonal are layered on top of one another to create a density gradient. Lipid encapsulated EVs applied to the bottom of the gradient float upwards during ultracentrifugation (200 000 × *g* overnight) according to their density allowing separation of EV populations from contaminating proteins.^65,71,72^ Whilst a useful technique for laboratory based research, ultracentrifugation lacks practicality in a clinical setting due to time intensive preparation, significant equipment needs and poor scope for high throughput scalability.

On the other hand, ultrafiltration techniques, such as tangential or sequential flow filtration, support rapid EV isolation from large volumes of cell culture supernatants or biological fluids. In tangential flow filtration, EV containing solutions flow over a membrane filter with a defined molecular weight cut-off (commonly 500 kDa) to permit small particles, such as proteins and liquid, to flow through whilst retaining EVs in the retentate. This process supports the concentration of large or small volumes of liquid whilst capturing EVs. However, the technique yields EVs with high protein contamination and there are concerns of the impact the filtration membrane might have upon EV integrity. Where high purity is required, tangential flow filtration would need to be used in conjunction with a second technique to improve purity, such as size exclusion chromatography.^73–76^ Alternatively, sequential filtration uses a combination of three filtration steps to isolate EVs devoid of contaminating proteins. First, dead end filtration is used to remove cells and debris. This is followed by tangential flow filtration to concentrate the sample and retain EVs as described above. Finally filtration occurs through a track edged membrane of increasing pore sizes (50–200 nm) to isolate and fractionate EVs by size.^76^

Size exclusion chromatography is an efficient chromatography technique that separates particles based on size. This has been adapted to separate and purify EVs from proteins in complex biological samples. When biological fluids such as blood plasma/serum is applied to a sepharose size exclusion column, differential exclusion causes EVs to elute first and separately from proteins which get trapped in the resin pores and only begin to elute in later fractions.^77^ However, size exclusion chromatography technology cannot efficiently separate EVs from lipoproteins of similar size when used to purify EVs from plasma or serum. For complete lipoprotein removal a mixture of isolation and purification methods would need to be carried out including density gradient ultracentrifugation followed by size exclusion chromatography.^68^ Other chromatography techniques that have been developed for EV purification include affinity purification and ion exchange chromatography. Membrane affinity purification methods such as exoEasy spin columns can isolate EVs from biological samples, however purity may be sub-optimal in comparison to size exclusion chromatography.^78^ Affinity based methods have also been optimized using the calcium sensitive, phosphatidylserine binding protein Tim4. EVs bound to Tim4 can subsequently be simply released by addition of calcium chelators.^79^ Other immuno-affinity capture agents can include heparin, tetraspanins and Epithelial Cell Adhesion Molecule (EpCAM).^80–82^ However the disadvantage of immuno-affinity capture is that only select populations of EVs are purified and EVs can be difficult to remove from the substrate without very harsh conditions such as low pH. A final chromatography method that is proving to be efficient for the isolation of EVs from cell culture supernatants in a scalable and efficient manner is anion exchange chromatography.^83,84^ Negatively charged EVs bind to positively charged columns and EVs are then eluted from the column using increasing concentrations of salt. Using anion exchange chromatography EVs can be isolated from 1 liter of cell culture conditioned media within 2 hours with minimal user input.^83^ This approach to EV isolation demonstrates scalability and rapid isolation suggesting it may have promise in supporting the use of EVs as therapeutics.

A popular method for isolation of EVs from clinical biological samples is by precipitation using commercially available reagents. The precipitation of EVs using polyethylene glycol (PEG),^85^ or commercially available reagents such as exoquick^86^ allows EVs to be pelleted by centrifugation at lower speeds, removing the need for time consuming and equipment dependent ultracentrifugation. EVs can be captured from small volumes of biological fluids, or larger volumes of pre-concentrated biological fluids/cell culture supernatants. Although very user friendly and amenable for use with a large number of biological samples, some reports suggest that EV purity after precipitation can be low as precipitation can also pellet proteins and lipoproteins.^86,87^ A second purification step after precipitation may be necessary to isolate a pure preparation of EVs. Additionally, precipitation reagents remaining in EV preparations can affect recipient cell viability EV and biological activity.^88,89^

Finally microfluidic chips are an emerging technology useful for the capture and analysis of EVs from small volumes of clinical samples and show promise for liquid biopsy diagnosis of disease. Microfluidic devices have been engineered for immuno-capture using tumor specific antigens or other markers of interest. For example, Human Epidermal Growth Factor Receptor (HER2) and Prostate Specific Antigen (PSA) positive tumor derived EVs have been captured on chips employing nanoshearing fluid flow. Captured EVs can then be quantified by a colorimetric reaction.^90^ Others have isolated and then eluted EVs for downstream analysis. For instance, EGFR wild type or EGFRvIII EVs can be isolated and quantified from glioblastoma patient plasma. Additionally EVs have been eluted from a chip and used for further in-depth RNA sequencing of EGFRvIII EVs.^91^ An alternative chip device approach used EpCAM aptamers to capture EVs, and electro-oxidation of metal nanoparticles to detect specific epitopes present upon captured EVs (specifically EpCAM and Prostate Specific Membrane Antigen (PSMA)). Oxidation of metal particles provides an electro chemical peak that can be used as a read out for quantification of captured EVs.^92^ Microfluidic chips have also been designed with specific size thresholds to capture tumor derived microvesicles. The EVs travel through the microfluidic chip and are eluted from different ports (dependent on size) for further downstream processing.^93^Table 1 summarizes the pros and cons of current popular EV isolation techniques.

**Table 1:** Pros- and cons-of different EV isolation techniques

Isolation	Pros	Cons
Ultracentrifugation	Well characterized and common technique. Isolates and separates large EVs from small EVs	Time consuming. Significant equipment needs. Contaminating proteins and lipoproteins are not removed
Gradient	Separates EVs from contaminant proteins and some lipoproteins	Time consuming. Significant equipment needs. Further purification steps may be needed for complete lipoprotein removal
Ultrafiltration	Rapid isolation of EVs from large volumes	Contaminating proteins/lipoproteins are not removed
Size exclusion chromatography	Rapid isolation of EVs from small volumes of biological samples or from larger volumes that have been pre-concentrated	High-throughput scalability low. Further purification needed for removal of lipoproteins
Affinity	Rapid isolation	Only isolates very specific EV populations. Difficult to remove from beads intact
Anion exchange chromatography	Rapid isolation of EVs from large volumes of cell culture media. Early evidence suggests high purity	Necessity of further purification steps is to be determined
Precipitation	Rapidly isolate EVs from biological samples. High-throughput scalability	Pre-concentration needed for large volumes. Further purification often needed to remove contaminating proteins and lipoproteins
Microfluidic chips	Rapid processing from small volumes of biological samples. High-throughput scalability	Engineering and fabrication of chips; not readily commercially available

### EV characterization

Equally as important as selecting a method for EV isolation and purification is the characterization of EVs before use in downstream assays. There is an array of different methods that can be used to validate EV size, concentration, purity, and biomarker presence. Western blotting is a standard method used whereby probing for different EV markers in conjunction with a biomarker of interest can be carried out. EV markers that can be used include CD63, CD81, CD9, ALIX, TSG101, Flotillin and Annexins amongst many others.^72^ These markers do not differentiate between different EV subtypes but do confirm the presence of EVs. Western blots can, to some extent, also be useful in the analysis of EV purity. Western blotting for contaminant proteins such as cell organelle specific proteins and albumin which should be absent from EV preparations, can be useful to determine the extent of protein contamination in the EV preparation.^72^ To analyze the concentration and size of EVs there are several technologies available such as nanoparticle tracking analysis (NTA) and tunable resistive pulse sensing.^94–97^ Although useful, these technologies cannot differentiate between EVs, protein aggregates or lipoproteins and as such quantify all small particles in a solution. Dynamic light scattering (DLS) can be used to determine the size distribution of EVs but not their concentration. Accurate data is dependent upon the use of monodisperse particulate solutions and again the technology cannot differentiate between EVs, proteins lipoproteins or other particulate matter.^98^ To determine EV preparation purity, a method has been developed whereby the calculated value from the ratio of EV particle concentration to protein concentration can be used to estimate EV preparation purity.^99^

To visualize EVs electron microscopy imaging is routinely performed to assess EV morphology and quality as well as any other co-isolated contaminates such as protein, DNA, lipoprotein, or virus.^100,101^ Immunogold electron microscopy can be used to stain EVs for EV markers or biomarker, typically a protein, of interest.^65^ EVs can also be analyzed using flow cytometry whereby EVs are adhered to magnetic/latex beads and stained using lipid dyes or antibodies to EV markers.^102,103^ More recently nanoscale flow cytometry technology has been developed which allows for analysis of individual EVs by staining them with antibodies against biomarkers of choice, without the need to adhere them onto beads. Nanoscale flow cytometry has shown great promise for analyzing EVs, particularly in complex biofluids, but is still in its infancy of development. Proper instrumentation, antibodies and controls must be used to ensure accurate EV detection.^104–109^

**Fig. 3:**
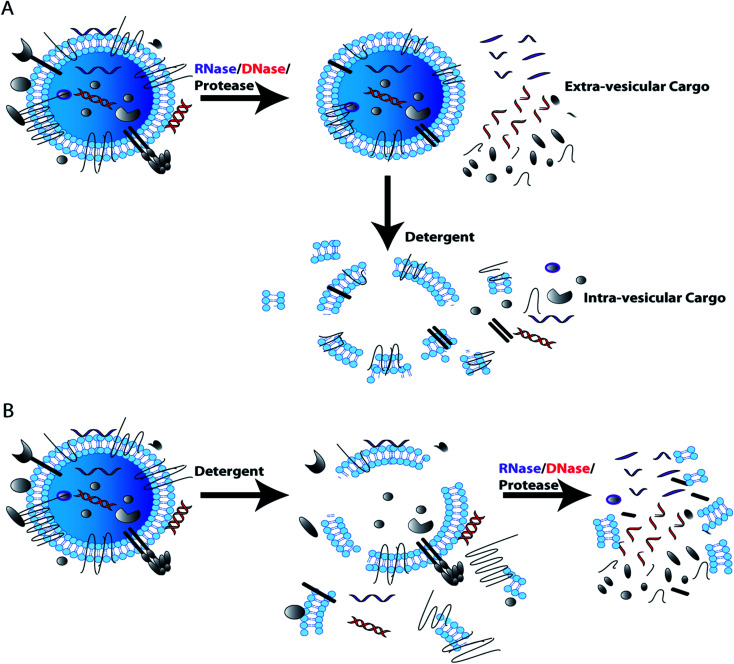
DNase, RNase, and protease use in EV membrane protection control experiment for EV cargo characterization. Assessment of cargo or biomarker EV association (intra-vesicular) from co-isolated contaminants (extra-vesicular), as performed through treatment with RNase, DNase or protease in the absence and presence of a lytic detergent. If cargo of interest is present post-RNase/DNase/protease treatment the cargo is likely intra-vesicular (A); additional studies using lysis followed by RNase/DNase/protease treatment further support findings (B). However, loss of cargo after RNase, DNase and protease treatment, indicates that cargo of interest is extra-vesicular and could be a contaminant that co-isolated with EVs.

In characterizing EV content, it is important to show that the RNA/DNA/protein of interest is truly EV associated and not simply co-purified with EVs. For example, albumin and lipoproteins are abundantly present in plasma and serum and co-isolate with EVs by most currently used isolation techniques.^68,69,86,87^ Soluble proteins and DNA associated with the outer EV membranes have also been shown to have utility in diagnostics. However, membrane associated particles may be more difficult to replicate in cross validation studies.^107,110–112^ To validate nucleic acids/proteins of interest are encapsulated by EV membranes, EVs can be treated with RNase and/or DNase or protease with and without tritonX-100/detergent lysis. If the cargo is detected when EVs are treated with RNase/DNase/protease, but are not detected if detergent lysis of EVs is followed by RNase/DNase/protease treatment, this indicates that the cargo resides within EVs and is protected by the EV membrane. If the cargo is not detected when intact EVs are treated with RNase/DNase, this indicates that the cargo is not protected by the EV membrane and is most likely an EV contaminant that co-isolated with EVs. Protease treatment of EVs will also remove transmembrane and membrane associated proteins from the EV surface as well as co-isolated contaminant proteins. Additional controls can support the validation of EV membrane proteins exclusively associated the external EV surface. For example immuno-gold electron microscopy enables direct visualization of protein localization within the EV preparation.^65^ These simple RNase/DNase/protease EV membrane protection control experiments can determine whether the cargo of interest is EV associated or an experimental contaminant (Fig. 3).^72^

Overall, the choice of EV isolation and characterization methods are very important for determining that the EVs being studied are *bona-fide* and pure. If EVs are impure – there is a chance that the results obtained in an experiment are not necessarily directly attributed to the isolated EVs but in fact due to co-isolated contaminants. This, in particular, could make cross validation studies difficult and reduce reproducibility of studies. Additionally if the EV concentration used is inaccurately reported then the data interpretation and comparison to other studies can be ineffective. Adherence of the scientific community to performing and reporting on EV characterization and control experiments in pre-clinical and clinical studies ensures that published EV data is accurate, easily interpreted, and reproducible.

## Extracellular vesicles and their clinical applications

### Pre-clinical cancer EV diagnostic and prognostic biomarker studies

Numerous studies have investigated EV number and EV protein or nucleic acid content as diagnostic and prognostic biomarkers in several different types of cancer. For instance, it has been demonstrated that elevated levels of circulating plasma EVs correlates with poor prognosis of colorectal cancer patients,^113^ whereas low levels of circulating plasma EVs is a poor prognostic indicator of esophageal cancer.^114^ More commonly investigated is the association of EV contents with cancer diagnosis and prognosis. For example, EV associated double stranded DNA fragments isolated from cancer cell culture supernatant and the blood of tumor bearing mice can be used to detect mutated oncogenes.^14^ Furthermore, in prostate cancer, EV associated DNA reflects gene copy number alterations that are commonly associated with prostate cancer metastasis and, interestingly, most of the EV associated DNA was exclusive to the large EVs/oncosomes.^59^

Instead of DNA, many research groups investigate the association of different RNA species present in EVs with cancer diagnosis and prognosis. Elevated levels of heterogeneous nuclear ribonucleoprotein H1 messenger RNA (mRNA) is detected in serum derived EVs of hepatocellular carcinoma patients^115^ and elevated human telomerase reverse transcriptase mRNA in circulating serum EVs has shown promise as a pan-cancer marker.^116^ RNA silencing microRNAs (miRNAs) are present in abundance inside EVs and many studies have found that miRNAs can act as diagnostic and/or prognostic indicators of several types of cancer. Pre-clinical studies have shown diagnostic and prognostic value of EV miRNAs in renal,^117^ non-small-cell lung,^118,119^ glioma,^120^ hepatocellular,^121,122^ ovarian,^123,124^ pancreatic^125,126^ and colorectal cancers^127,128^ to name only a few. Additionally, EV associated long non-coding RNAs (lncRNA) are also proving useful for cancer diagnosis and prognostication in pre-clinical studies. EV associated lncRNA-p21 is elevated in the urine of prostate cancer patients and can distinguish between benign disease and cancer.^129^ Plasma EV associated lncRNA UEGC1 shows promise as a diagnostic for early stage gastric cancer,^130^ and serum derived EV-associated HOTTIP may be useful as a diagnostic and prognostic indicator of gastric cancer.^131^ Finally, lncRNA MALAT1 is elevated in serum EVs from non-small-cell lung cancer patients.^132^ The less common family of circular RNAs is becoming increasingly recognized to be associated with EVs and potentially useful as a cancer biomarker.^133^ In fact, the presence of circular RNA-PDE8A is associated with pancreatic cancer diagnosis and progression.^134^

Finally, proteins either encapsulated by or associated with EV membranes can also be used for biomarker discovery. For example, circulating EV associated Copine III has been found to have diagnostic and prognostic significance for colorectal cancer.^135^ Melanoma biomarkers MIA and S100B are found on serum EVs and are predictive of diagnosis and prognosis of melanoma.^136^ Ascites derived EV associated *E*-cadherin has diagnostic utility in ovarian cancer and serum EV associated EphrinA2 may aid diagnosis of prostate cancer.^137,138^

There is a large number of research studies published regarding the analysis of EVs as biomarkers for cancer. To assess the EV isolation and characterization methods employed by the large number of EV studies, we selected studies based on a previous meta-analysis study that looked at the clinical significance of EVs in cancer.^139^ This study narrowed down the available selection of cancer EV biomarker literature between 2010–September 2018. The authors did a literature search using the key words ‘exosome and cancer and diagnosis or prognosis’. The authors then reviewed all literature and included only studies that involved EVs in cancer patient biofluids with at least 10 patients and matched controls being used. The biomarker had to have clinical significance and reporting of specificity and sensitivity for diagnostic markers, and confidence interval reported for prognostic markers. The literature search was narrowed down to include 60 studies. We further narrowed these studies down to only include studies that used at least 20 patient samples and matched controls. We then used the identical search terms and found that since September 2018 an additional 448 reports have been published. Using the same parameters but increasing the sample size required to *n* ≥ 20 patient samples and *n* ≥ 20 matched controls, we narrowed this down further and identified an additional 38 studies into our analysis. We use a total of 88 studies here to determine the EV characterization and isolation methods commonly used in pre-clinical biomarker discovery studies (Table 2).

**Table 2:** Summary of the methods used in pre-clinical cancer diagnostic and prognostic biomarker studies to isolate and characterize EVs. UC: ultracentrifugation. EM/imaging: electron microscopy/any other method used to visualize EVs. NTA/DLS: nanoparticle tracking analysis/dynamic light scattering (or any other method used to measure particle size and/or concentration). WB: western blot. Flow: flow cytometry. Protein: protein concentration determination. RNase/DNase/protease: use of EV membrane protection control experiments

EV pre-clinical cancer biomarkers													
Biomarker (type)	Biofluid	EV isolation				EV characterization							Reference
		UC	Gradient	Precipitation	Other	EM/imaging	NTA/DLS	WB	Flow	Protein	RNase/DNase/protease	Other	
HOTTIP (lncRNA)	Serum			✓		Y	Y	Y		Y			140
miR-30c-5p (miRNA)	Urine	✓				Y	Y	Y					141
LINC02418 (lncRNA)	Serum			✓		Y	Y	Y			Y		142
miRNA-320d (miRNA)	Serum	✓				Y	Y	Y					143
Hsa-circ-0065149 (circRNA)	Plasma		✓										144
miR-1246 (miRNA)	Serum			✓		Y		Y					145
miR-21 & MMP1 (miRNA & protein)	Urine				✓	Y		Y					146
miR-181b-5p (miRNA)	Ascites			✓									147
miR-21& miR-92a (miRNA)	Plasma	✓				Y							148
miR-19b1-5p, 21-5p, 136-5p, 139-5p, 210-3p (miRNAs)	Urine				✓								149
GNAQ-6:1 (lncRNA)	Serum				✓	Y	Y	Y		Y			150
GPC-1 (protein)	Serum			✓	✓								151
Hsa-circ-0004771 (circRNA)	Serum			✓		Y	Y	Y		Y			152
miR-150-5p & miR-99b-5p (miRNA)	Serum	✓				Y	Y	Y			Y		153
Pcsk2-2:1 (lncRNA)	Serum			✓		Y	Y	Y					154
5 lncRNAs (lncRNA)	Serum			✓		Y	Y	Y					155
4 miRNAs (miRNA)	Serum			✓							Y		156
H19 (lncRNA)	Serum			✓		Y	Y	Y		Y			157
lncSLC2A12-10:1 (lncRNA)	Plasma			✓		Y	Y	Y		Y			158
8 miRNAs (miRNA)	Plasma			✓		Y		Y	Y	Y	Y		159
miR-1910p-3p (miRNA)	Serum			✓		Y	Y			Y			160
Circ-PNN (circRNA)	Serum			✓		Y	Y	Y		Y			161
TBILA & AGAP2-AS1 (lncRNA)	Serum			✓		Y	Y	Y		Y	Y		162
miR-210 (miRNA)	Serum	✓				Y							163
HULC (lncRNA)	Serum			✓									164
miR-320d (miRNA)	Serum			✓				Y					165
CEBPA-AS1 (lncRNA)	Plasma	✓									Y		166
miR-378 (miRNA)	Serum			✓									167
miR-874 (miRNA)	Serum			✓									168
miR-10b-5p (miRNA)	Serum			✓		Y							169
8 miRNAs (miRNA)	Ascites	✓					Y						170
miR-17-5p (miRNA)	Serum			✓		Y	Y	Y					171
GAS5 (lncRNA)	Serum			✓		Y	Y	Y		Y			172
FGB & FGG (protein)	Plasma				✓	Y		Y		Y			173
*c-MET & PDL1 (protein)	Serum			✓									174
H19 (lncRNA)	Serum			✓		Y	Y	Y			Y		175
miR-4525, miR-451a& miR-21 (miRNA)	Plasma	✓				Y							176
PCAT-1, UBC1 & SNHG16 (lncRNA)	Serum			✓		Y	Y	Y		Y	Y		177
miR-454-3p (miRNA)	Serum			✓									178
alpha-2-HS-(glycoprotein) & extracellular matrix protein 1 (protein)	Serum	✓				Y	Y	Y		Y			179
miR-210 (microRNA)	Serum			✓		Y		Y					180
16 lipids (lipid)	Plasma	✓					Y						181
PTENP1 (lncRNA)	Plasma			✓		Y		Y		Y	Y		182
KRAS (DNA)	Plasma	✓											183
miR-122, miR-125b, miR-145, miR-192, miR-194, miR-29a, miR-17-5p, and miR-106a (miRNA)	Serum			✓		Y		Y					184
lncRNA PRINS (lncRNA)	Serum			✓									185
miR-200b (miRNA)	Plasma			✓				Y		Y			123
Copine III (protein)	Plasma	✓				Y	Y	Y		Y			135
miR-122-5p, miR-125b-5p, miR-192-5p, miR-193b-3p, miR-221-3p and miR-27b-3p (miRNA)	Plasma			✓									186
TACSTD2 (protein)	Urine	✓				Y		Y	Y	Y			187
ENST00000													
588480.1/517758.1 (lncRNAs)	Bile	✓				Y	Y		Y				188
CRNDE-h (lncRNAs)	Serum			✓		Y		Y		Y	Y		189
miR-21 (miRNAs)	Serum			✓									190
91H (lncRNAs)	Serum			✓		Y		Y		Y			191
miR-4772-3p (miRNA)	Serum			✓		Y	Y	Y					128
miR-19a (miRNA)	Serum	✓		✓		Y							127
miR-548c-5p (miRNA)	Serum			✓									192
miR-200 family (miRNA)	Plasma	✓				Y		Y					193
miRNA-21 (miRNA)	Plasma	✓				Y							194
miR-6869-5p (miRNA)	Serum			✓									195
miR-6803-5p (miRNA)	Serum			✓									196
EVs	Plasma			✓			Y					Y	114
lncUEGC1 (lncRNA)	Plasma	✓	✓			Y	Y	Y		Y	Y		130
miR-423-5p (miRNA)	Serum			✓		Y	Y	Y		Y			197
miR-23b (miRNA)	Plasma	✓				Y							198
miR-451 (miRNA)	Serum			✓									199
RNU-1 (sncRNA)	Serum			✓		Y	Y	Y	Y		Y		200
miR-301a (miRNA)	Serum			✓						Y			120
miR-21 (miRNA)	Serum			✓									201
hnRNPh1 (mRNA)	Serum			✓									115
ENSG00000258332.1 & LINC00635 (lncRNA)	Serum			✓									202
miR-125b (miRNA)	Serum			✓		Y	Y	Y		Y			121
miR-638 (miRNA)	Serum			✓									122
miR-93 (miRNA)	Serum			✓						Y			203
LINC00161 (lncRNAs)	Serum			✓		Y	Y	Y		Y			204
miR21 (miRNA) & HOTAIR (lncRNA)	Serum			✓		Y		Y		Y			205
MIA & S100B (protein)	Serum			✓			Y	Y		Y			136
MALAT-1 (lncRNAs)	Serum			✓		Y	Y	Y		Y			132
miR-451a (miRNA)	Plasma	✓				Y							119
miR-373, 200a, 200b & 200c (miRNAs)	Serum			✓				Y				Y	124
miR-451a (miRNA)	Plasma	✓				Y							206
miR-191, 21 & 451a (miRNAs)	Serum			✓									125
Glypican-1 (proteoglycan)	Serum	✓	✓			Y	Y	Y		Y			207
miR-125b-5p (miRNA)	Plasma			✓									186
p21 (lncRNAs)	Urine				✓								129
EphrinA2 (protein)	Serum	✓				Y		Y		Y			137
SChLAP-1 (lncRNAs)	Plasma			✓		Y	Y	Y		Y			208
miR-1290 & 375 (miRNA)	Plasma			✓									209
	**Total: 88**	**23**	**3**	**60**	**6**	**52**	**34**	**45**	**4**	**31**	**12**	**2**	

Analysis of the EV isolation and characterization techniques used by 88 pre-clinical biomarker discovery studies revealed that the most common EV isolation method used is precipitation (60/88) and ultracentrifugation (23/88). Whilst ultracentrifugation is not a scalable or time efficient technique to use in a diagnostic clinical setting, EV precipitation is an excellent technique to use for isolation of EVs from large numbers of small volume biological fluids. However, due to reported impurities of EV preparations after some precipitation techniques,^86,87^ careful characterization of EVs needs to be done to ensure the biomarker of interest is actually EV associated. The most popular EV characterization methods used by this set of studies was electron microscopy/imaging (52/88), followed by western blot for EV markers (45/88), protein concentration determination (31/88) and nanoparticle tracking analysis/dynamic light scattering (34/88). The Minimal Information for Study of Extracellular Vesicles (MISEV) 2018 guidelines suggest that studies should carry out the following characterization steps: analysis of single EVs by two techniques (such as electron microscopy/imaging AND NTA/DLS) evaluation of EV marker and contaminant proteins (western blot/flow analysis), quantification of EV preparation (protein concentration/protein concentration : particle concentration ratio for example).^72^ Most studies carried out at least one or more of the EV characterization techniques but only 18 completed all recommended characterization steps. Surprisingly, there were 27 studies out of 88 that did not do any form of EV characterization. For some studies this may be due to previous publications reporting the EV isolation and characterization. Even in these cases it would still be beneficial to provide some quality control EV characterization in their current publications and clearly direct the reader to previous publications on characterization studies. Very few (12/88) of the analyzed studies included an RNase, DNase or protease EV membrane protection control experiments to determine whether the EV associated cargo/biomarker was in fact EV enclosed/associated and not a co-isolated contaminant. During the discovery phase of biomarker development, this is an important control experiment to include to present clear data that could be used to move forward to a clinical setting.

### Clinical translation of EV diagnostic and prognostic biomarker studies in cancer

While many studies have investigated new cancer biomarkers in pre-clinical settings, there are only a handful of biomarker tests that have been current good manufacturing practice (cGMP) approved and only one has been approved for use by clinicians (Table 3). Exosome Diagnostics, Inc. has developed workflows to isolate cell free DNA and EV associated DNA/mRNA from plasma using a cGMP clinically certified process Exolution™ Plus. The DNA and EVs are then subject to lysis and quantitative RT-PCR (qRTPCR) analysis of EGFR mutations. This has clinical utility for lung cancer patients to direct patient treatment strategies.^210–212^ The same company has developed an additional cGMP and clinically approved technology – ExoPro urine clinical sample concentrator kit – to isolate EVs from urine by ultrafiltration centrifugation and to extract EV associated RNA. QRTPCR was used to analyze the expression of a set of genes (PCA3, SPDEF, ERG) and found to have utility as non-invasive test to risk stratify men with suspected prostate cancer and improve the identification of individuals with clinically significant disease.^213–215^

**Table 3:** Table summarizing the EV diagnostic tests that are in the clinic or cGMP certified

Clinical/cGMP approved cancer EV biomarker studies				
Biomarker	Isolation	Characterization	Test	In clinic?
EGFR mutation in NSCLC in plasma^217^	Exolution™ plus: isolated EV and cell free DNA. cGMP	WB, NTA, SEM characterization^218^	qRTPCR for EGFR mutations	No
EGFR T790M NSCLC in plasma^212^	Exolution™ plus: isolated EV and cell free DNA cGMP	WB, NTA, SEM characterization^218^	qRTPCR for EGFR mutations	No
EGFR activating/resistance mutation detection NSCLC in plasma^210^	Exolution™ plus: isolated EV and cell free DNA. cGMP	WB, NTA, SEM characterization^218^	qRTPCR for EGFR mutations	No
ExoDX™ prostate intelliscore in urine^213–215^	EXOPRO urine clinical sample concentrator kit. cGMP	Characterized in pre-clinical studies	qRTPCR	Yes

Why are there so many pre-clinical studies identifying new cancer biomarkers and so few are getting to clinic? There are several reasons why a bottle neck exists. These include lack of implementation of robust methodologies that comply with good laboratory practice (GLP) and cGMP protocols that are also approved for use in the clinic.^216^ Currently, Exosome Diagnostics, Inc. uses their cGMP and clinical laboratory approved technologies to isolate EVs from prostate cancer patient urine and non-small cell lung carcinoma patient plasma. In order to reduce the bottle neck, there is a need for the development of a simple EV isolation protocol that is cGMP approved and can be used widely in a clinical setting to support the analysis of multiple clinical samples in a high through put manner. Whilst reagents have been developed that are commonly used in pre-clinical biomarker discovery studies and are also GLP and cGMP compliant, such as exoquick (EV precipitation reagent), there is conflicting information in the literature regarding the efficiency of precipitation in isolating pure EV preparations from biological samples.^78,86–88^

### Pre-clinical development of EV therapeutic delivery vehicles for cancer

Many research groups are developing therapeutic EVs that can be used as delivery vehicles of small molecules (RNA, DNA, proteins and pharmacological agents) for cancer treatment (Table 4). EVs loaded with small molecules can deliver chemotherapeutics such as paclitaxel and doxorubicin to cancer cells and reduce off target effects on non-specific organs.^219–222^ Cargo loading of small molecules can be achieved through manual loading of EVs post-EV isolation by incubation, electroporation or sonication. It has also been demonstrated that cells can be engineered to load oncolytic virus into EVs. EVs loaded with oncolytic virus and paclitaxel increased antitumor effects both *in vitro* and *in vivo* as compared to paclitaxel loaded EVs alone.^223^ For these systems, EV contaminating proteins must be assessed in addition to an assessment of efficient removal of excess drug; this is essential to allow accurate control of dosage and drug delivery.

**Table 4:** EV isolation and characterization methods used in pre-clinical studies generating EVs as therapeutic delivery vehicles. DC/UC: differential centrifugation/ultracentrifugation. UF: ultrafiltration. Gradient/SC: gradient/sucrose cushion. SEC: size exclusion chromatography. EM/imaging: electron microscopy/any other method used to visualize EVs. NTA/DLS: nanoparticle tracking analysis/dynamic light scattering (or any other method used to measure particle size and/or concentration). WB: western blot. Flow: flow cytometry. Protein: protein concentration determination. RNase/DNase/protease: use of EV membrane protection control experiments. N/A: not applicable

EVs as therapeutic delivery vehicles													
Cargo (target/drug)	EV isolation					EV characterization							Reference
	DC/UC	UF	Gradient/SC	Precipitation	SEC	EM/imaging	NTA/DLS	WB	Flow	Protein	DNase/RNase/protease	Detergent	
Small molecule (doxorubicin)	✓	✓	✓			Y	Y				N/A		219
Small molecule (doxorubicin)		✓					Y	Y		Y	N/A		220
Small molecule (Piclitaxel)	✓				✓	Y	Y	Y		Y	N/A		221
Small molecule (piclitaxel)				✓	✓	Y	Y	Y		Y	N/A		222
Oncolytic virus and piclitaxel	✓						Y				N/A		223
siRNA (Kras)	✓		✓			Y	Y		Y	Y	RNase, proteinase K	Y	224
siRNA (Rad51, Rad52)	✓					Y	Y	Y		Y			225
siRNA (PLK-1)	✓									Y			226
miRNA & siRNA (Let7, VEGF)		✓		✓		Y	Y	Y			RNase		227
miRNA (mir-134)	✓			✓		Y		Y		Y	RNase		228
miRNA (mir-31, mir-451a)	✓					Y	Y	Y	Y	Y	RNase	Y	240
gRNA (reporter)		✓			✓	Y	Y	Y		Y	RNase, proteinase K	Y	231
DNA (Cas9/gRNA – RUNX2 & CTNNB1)				✓		Y	Y	Y		Y	DNase, proteinase K		233
DNA (Cas9/gRNA – PARP1)		✓		✓		Y	Y	Y		Y	DNase		232
Protein & gRNA (Cas9/gRNA RNP – HIV LTR)	✓						Y			Y			234
Protein/mRNA (p53/Cas9 gRNA RNP)	✓		✓			Y	Y	Y					238
Protein (Bax/srlkB/cre)	✓			✓			Y	Y		Y			237
Protein (ww-cre)	✓		✓				Y	Y		Y	Proteinase K	Y	239
Total: 18	**12**	**5**	**4**	**6**	**3**	**12**	**16**	**13**	**2**	**14**	**8**	**4**	

EVs can be loaded with small interfering RNA (siRNA) and miRNA to manipulate gene expression in cancer cells and reduce tumorigenic phenotypes. For example, siRNA to kRAS^G12D^ has been packaged into foreskin fibroblast-derived EVs by electroporation. Intraperitoneal injection of kRAS^G12D^ siRNA loaded EVs resulted in regression of not only the primary pancreatic tumor, but also the metastatic tumors.^224^ Loaded EVs delivering siRNA against Rad51 (ref. 225) and PLK1 (ref. 226) to cancer cells *in vitro* reversed cancer phenotypes. In another example, EVs were both targeted to nucleoilin (which is over-expressed on breast cancer cell membranes) and loaded with VEGF siRNA and Let-7 miRNA to initiate anti-tumor activity *in vitro* and *in vivo*.^227^ Rather than electroporation, another study used engineered cell lines to overexpress miRNAs of interest which passively load into EVs during biogenesis.^228^

An alternative way to manipulate cancer cell gene expression is to edit the genome directly using CRISPR/Cas9.^229,230^ This technology has the potential to treat any disease with a genetic basis including cancer, however, it requires an appropriate vehicle for its delivery to diseased cells. Guide RNA can be loaded into EVs using simple over-expression and delivered in a functional capacity to recipient reporter cells *in vitro*.^231^ Other methods have loaded DNA plasmids encoding both the Cas9 protein and the guide RNA into isolated EVs by electroporation^232^ or lipofectamine hybridosome formation^233^ to deliver gene editing machinery into cancer cells and mesenchymal stem cells, respectively. Alternatively, Cas9 protein forms a ribonuclease protein complex (RNP) with guide RNA, this RNP can be loaded into EVs by rapalog induced dimerization of two fusion proteins: membrane associated mCherry picker-DMRA and Cas9-DMRC. Co-expression of vesicular stomatitis virus G protein (VSVg) induces budding of a unique form of EVs called ‘Gesicles’. Rapalog induced dimerization of DMRA and DMRC localizes Cas9-gRNA RNP to the membrane in the vicinity of EV biogenesis ensuring its incorporation into the specialized Gesicles.^234^ Alternative dimerisation systems have also been designed and used to load Cas9-gRNA into EVs.^235,236^

**Fig. 4:**
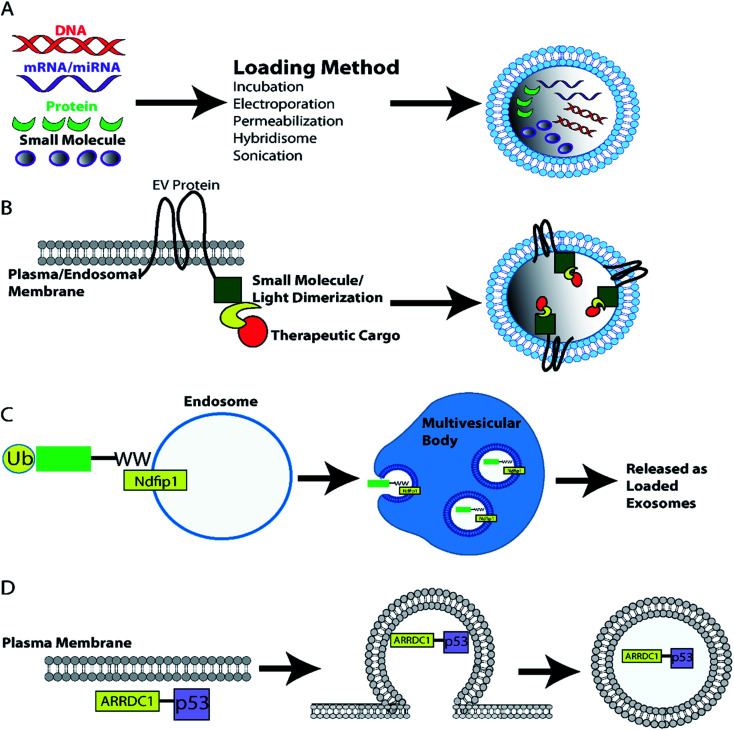
EV loading with therapeutic cargoes. (A) EVs can be loaded with therapeutic cargoes post-isolation using techniques such as incubation, electroporation, sonication, hybridization with lipofectamine, and permeabilization. Alternatively, cell lines can be engineered to load EVs with protein cargoes during biogenesis. (B) Generation of EV marker and cargo fusion proteins enables light/ligand induced dimerization loading of EVs as dimerization localizes the desired cargo to the plasma/endosomal membrane – the site of EV biogenesis.^234,237^ (C) Alternatively WW-tagged proteins promotes loading of ubiquitinated cargo into endosomal intraluminal vesicles and eventual release as exosomes.^239^ (D) Furthermore, the ARRDC1-p53 fusion protein recruits ESCRT machinery to the plasma membrane inducing p53 filled EV ARMM budding from the plasma membrane.^238^

Another heterodimerisation system uses light to load protein cargo into EVs. The two fusion proteins, CD9-CIBN and cargo-Cry2, dimerize in the presence of blue light. Dimerization of cargo protein to membrane localized tetraspanin CD9, leads to incorporation of cargo protein into EVs during biogenesis. This system can deliver proteins such as tumor suppressor Bax, Srlkb and Cre to recipient cells *in vitro* and *in vivo*.^237^ Another study used Arrestin domain containing protein 1 (ARRDC1) which localizes to the plasma membrane (cytosolic side) and recruits ESCRT machinery to initiate ARRDC1-mediated microvesicle budding (ARMMs). Fusion of tumor suppressor p53 with ARRDC1 led to incorporation of functional ARRDC1-p53 into ARRMS.^238^ Finally, tagging of cargo proteins such as Cre with a WW-tag drives the proteins ubiquitination and localization to endosomal membranes where WW-tagged proteins are incorporated into intraluminal vesicles during exosome biogenesis. Exosome loaded WW-Cre was functionally delivered *in vivo*.^239^ The various approaches that can be used to load therapeutic cargoes into EVs both pre- and post-isolation are summarized in a diagram in Fig. 4.

Diverse types of cargo have been loaded into EVs and the techniques/strategies used vary from study to study. Table 4 details the cargo loading approaches used in pre-clinical therapeutic EV studies and the EV characterization techniques performed. The majority of studies used differential ultracentrifugation to isolate EVs (12/18). The second most common technique used to isolate EVs was EV precipitation reagents such as exoquick or PEG, or in one case immunoprecipitation (6/18). In some studies that used precipitation, a second EV purification procedure was carried out. The majority of studies that did not use a second purification technique after precipitation included RNase/DNase/protease EV membrane protection control experiments. Less than half of the studies characterized isolated EVs with imaging, NTA/DLS, protein quantification AND western blot or flow cytometry for EV markers (8/18), and 4/18 used only one or two of the MISEV recommended EV characterization techniques. Over half of the eligible reviewed studies carried out, RNase/DNase/protease EV membrane protection control experiments (8/13), and 4 studies use detergent lysis of EVs in conjunction with the EV membrane protection control experiment. These control experiments are important to ensure that the therapeutic cargo of interest is incorporated within the EV lumen and not delivered to recipient cells as a co-isolated contaminating protein or nucleic acid.

### Clinical translation of EVs as therapeutic vehicles for cancer

Clinical trials have been conducted using dendritic-cell derived exosomes (Dex) pulsed with tumor antigens to stimulate an anti-tumor immune response.^241,242^ Second generation Dex derived from interferon (IFN)-γ-maturated dendritic cells (IFN-γ-Dex) have also been used in a phase II clinical trial on non-small cell lung cancer (NSCLC) patients.^243^ As an alternative approach, EVs derived from tumor ascites have been used in clinical trials for immunotherapy in colorectal cancer.^244^ To date, these studies represent the majority of published clinical trials that have used EVs as therapeutic delivery vehicles to treat cancer. There are many studies in progress/recruiting, the results of these will be expected over the coming years.

The use of EVs as therapeutic delivery vehicles requires large scale manufacturing of clinical grade EV doses. Several papers have been published describing safe GMP of clinical grade EVs (Table 5). In the first protocol developed in 2002, monocyte derived dendritic cell conditioned media was filtered to remove cells and debris and concentrated using tangential flow filtration (TFF) with a 500 MWCO hollow fiber membrane. EVs were then purified using sucrose density cushion and ultracentrifugation and finally diafiltrated into a physiological buffer using TFF and filter sterilized using a 0.22 μm filter pore size. The use of TFF in the protocol allows for processing of large volumes of cell culture supernatant in a time efficient manner and the multi-step process maximizes the purity of EVs.^75^ This protocol has been commonly used and adapted in subsequent clinical trials^241–243^ and GMP development studies.^245^ However, both the original and subsequent studies did not carry out extensive EV characterization and relied predominantly upon ELISA and flow cytometry.

**Table 5:** Summary of EV isolation and characterization techniques used in clinical/cGMP approved therapeutic EV studies. DC/UC: differential centrifugation/ultracentrifugation. UF: ultrafiltration. SEC: size exclusion chromatography. EM/imaging: electron microscopy/any other method used to visualize EVs. NTA/DLS: nanoparticle tracking analysis/dynamic light scattering (or any other method used to measure particle size and/or concentration). WB: western blot. ELISA: enzyme linked immunosorbent assay. Protein: protein concentration determination. Flow: flow cytometry. RNase/DNase/protease: use of EV membrane protection control experiments. Detergent: use of EV membrane protection control experiment in conjunction with detergent lysis of EVs

EVs as therapeutic vehicles in the clinic															
EV	EV purification					EV characterization								Reference	Clinical phase
	DC/UC	UF	Gradient	Precipitation	SEC	EM/imaging	NTA/DLS	WB	ELISA	Protein	Flow	DNase/RNase/protease	Detergent		
Dendritic cell derived EVs		✓	✓						Y	Y	Y			75	GMP^a^
Dendritic cell EVs		✓	✓						Y					241	Phase, 1 CT^a^
Colorectal cancer ascites EVs	✓		✓			Y		Y		Y				244	Phase, 1 CT
Bone marrow derived MSC EVs	✓					Y	Y			Y	Y	RNase,	Y	248	GMP
Mesenchymal stromal cell derived EV	✓						Y	Y		Y		RNase, proteinase K		247	GMP
Hek293 EVs	✓	✓			✓	Y	Y	Y	Y	Y	Y			246	GMP
Dendritic cell derived EVs		✓	✓						Y					242	Phase, 1 CT^a^
Dendritic cell derived EVs		✓	✓							Y	Y			243	Phase, 2 CT^a^
Total: 8	**4**	**5**	**5**	**0**	**1**	**3**	**3**	**3**	**4**	**6**	**4**	**2**	**1**		

a Studies used protocols formulated/adapted from Lamparski *et al.*, 2002.^75^

Another study used a multi-step process to generate GMP compliant EV preparations from HEK293 cells. This study used a combination of filtration, TFF and size exclusion chromatography to generate GMP compliant EVs.^246^ The EVs were also thoroughly characterized using EM, ELISA, western blot and flow cytometry. Other GMP compliant EV purification studies generated clinical grade EVs using ultracentrifugation and/or gradients.^244,247,248^ The problem with ultracentrifugation and gradients is that they are time consuming and not as scalable as other techniques in the clinical setting if large numbers of EV doses are required to be manufactured. Overall 5/8 studies used ultrafiltration and 8/8 used ultracentrifugation and/or gradient.

Compared to pre-clinical studies, thorough characterization of EVs in most clinical studies is minimal. Only 3/8 studies showed EV structure by electron microscopy and 3/8 characterized particle concentration and size by NTA/DLS. However, 7/8 characterized EVs by western blot/flow, 4/8 used ELISA and 6/8 quantified protein. None of the studies carried out all characterization steps recommended by MISEV guidelines.^72^ Additionally, the RNase/DNase/protease EV membrane protection control experiments are rarely carried out (2/8). Although these studies may have been following protocols that have previously been developed and optimized, researchers should consider including more EV characterization and quality control datasets within clinical and cGMP compliant studies and/or provide clear reference to the publication that contains the EV characterization.

The majority of clinical studies using EVs as therapeutics use autologous primary human cells however some GMP studies have explored the purification of clinical grade EVs from cell lines. This is a stark contrast to pre-clinical studies where the majority of studies use human cell lines to generate therapeutic delivery vehicles that can be used *in vitro* and *in vivo*. The use of autologous EVs in pre-clinical research is time consuming and costly. Clinical trials show a clear preference for the use of autologous EVs to prevent any immunogenic side effects. However, current research suggests that cell line derived EVs are non-toxic and non-immunogenic *in vivo*.^63,64^ Further research needs to be conducted to understand the safety and clinical applicability of EVs derived from immortalized cell lines.

## The need for standardization of extracellular vesicle isolation and characterization protocols for clinical applications

Our review of the literature investigates the most common EV isolation and characterization techniques that are used by pre-clinical and clinical studies. The review has uncovered important points regarding what we should be doing as a scientific community when isolating and characterizing our EVs.

Identification of the optimal EV isolation protocol for pre-clinical studies is essential and dependent upon the fluid and volume from which EVs are being isolated. We conclude that it is possible to use any isolation technique deemed necessary (most frequently used was ultracentrifugation and precipitation) as long as characterization steps are employed: electron microscopy (or alternative), western blot/flow for EV markers and contaminants, EV preparation quantitation (protein concentration quantification for example) and nanoparticle tracking analysis (or alternative technology). In addition, the number of studies employing RNase/DNase/protease EV membrane protection control experiments were surprisingly low. Performing these simple experiments, would provide strong evidence that the biomarker being investigated or the therapeutic cargo loaded is in fact EV-associated/enclosed. Thorough EV characterization and EV membrane protection control experiments are important for the identification of *bona fide* EV cargoes; particularly given that other particles in biological fluids likely reside in EV preparations. These other particles may also have biomarker utility as has been shown for the extracellular particle termed exomere.^249,250^ Exomeres lack a lipid bilayer and likely arise through a distinct and different biogenesis pathway from EVs. Exomeres have a physiological role and have demonstrated potential as biomarkers for cancer.^249,250^ It is likely that all extracellular particles (such as EVs and exomeres) have potential to support the development of new diagnostic and prognostic testing. However, thorough characterization of the particle that one is working with will improve our understanding of the cargoes that they carry and the biological functions that they may have, enhance our understanding of small particles, and support study reproducibility.

For EV isolation in a clinical setting more cGMP approved technologies for small scale and large scale EV isolation need to be developed. Large scale isolation of EV therapeutic delivery vehicles in the clinic use multi-step methods to isolate pure EVs. However most methods include an ultracentrifugation and/or gradient step which are not easily scaled up and therefore these methods may not be suitable for large scale isolation of EV therapeutics in the clinic. Ultrafiltration technologies such as TFF can concentrate and isolate EVs at a large scale, but a second purification step is necessary. Therefore, other EV purification strategies that can isolate EVs in a large scale, quickly with minimal hands on input need to be developed. EV characterization in clinical studies often relies upon past publications using the same or similar isolation strategy. Improved reporting on past study EV characterization and inclusion of EV quality control and characterization would be beneficial in clinical studies.

## Conclusion

Is there a need for EV purification and characterization standardization? One could argue that there is not a need for standardization of EV purification protocols for pre-clinical and clinical studies, as long as purified EVs are thoroughly characterized and controlled. There is plenty of room for technology development in terms of EV isolation and purification for biomarker analysis and for therapeutic delivery vehicle development. MISEV recommend that EVs are characterized on the single molecule level using two techniques (electron microscopy/other imaging techniques and by a particle analysis technology such as NTA/DLS), analyzed for EV markers and absence of contaminants (western blot/flow cytometry), and that the EV preparation should be quantitated (protein concentration or protein concentration to particle number ratio).^72^ Currently a large proportion of pre-clinical and clinical studies still do not fully characterize the isolated EVs. EV characterization should be standardized and become routine within the field. Additionally, the use of the RNase/DNase/protease EV membrane protection control experiment should also become a new routine standard control experiment included in all EV biomarker and therapeutic loading studies. Without these simple controls, there is no way the reader can know if the cargo/biomarker of interest reported by the study is actually EV associated or is rather a co-isolated contaminant.

## Author contributions

NS and KCW conceptualized review. NS drafted the manuscript. NS and KCW reviewed and edited the manuscript. KCW generated manuscript figures.

## Conflicts of interest

There are no conflicts of interest to declare

## Supplementary Material
